# Big Five Development From Young Adulthood to Midlife: Findings From a Longitudinal Study of Mexican-Origin Adults

**DOI:** 10.1093/geroni/igab046.2155

**Published:** 2021-12-17

**Authors:** Olivia Atherton, Angelina Sutin, Antonio Terracciano, Richard Robins

**Affiliations:** 1 Northwestern University, Chicago, Illinois, United States; 2 Florida State University College of Medicine, Tallahassee, Florida, United States; 3 Florida State University, Florida State University, Florida, United States; 4 University of California, Davis, Davis, California, United States

## Abstract

A large body of research has documented how personality develops across adulthood, yet very little longitudinal work has examined whether these findings generalize beyond predominantly middle-class, highly-educated White American or Western European individuals. This pre-registered study uses longitudinal data from 1,110 Mexican-origin adults who completed a well-validated personality measure, the Big Five Inventory, up to 6 times across 12 years (median age at Wave 1 = 37.7; range = 26 to 65). Individuals generally maintained their rank ordering on the Big Five over time (rs=.66-.80), and all of the Big Five traits showed small, mean-level decreases across adulthood. These trajectories had few associations with sociodemographic factors (sex, education level, IQ) and cultural factors (generational status, age at immigration, Spanish/English language preference, Mexican cultural values, American cultural values, ethnic discrimination). Divergences between the present findings and previous research highlight the need to study personality development across diverse aging samples.

